# Supplementary material for: Splice-Junction-Based Mapping of Alternative Isoforms in the Human Proteome
Source: Cell Rep. Author manuscript; Available in PMC 2020 Jan 15. (PMC6961840; doi:10.1016/j.celrep.2019.11.026)
Supplement: 3 [file NIHMS1546469-supplement-3.zip › DF2/PXD006675/RightVentricle-341-Q2M3C7-SMSELVEEKK.pdf]

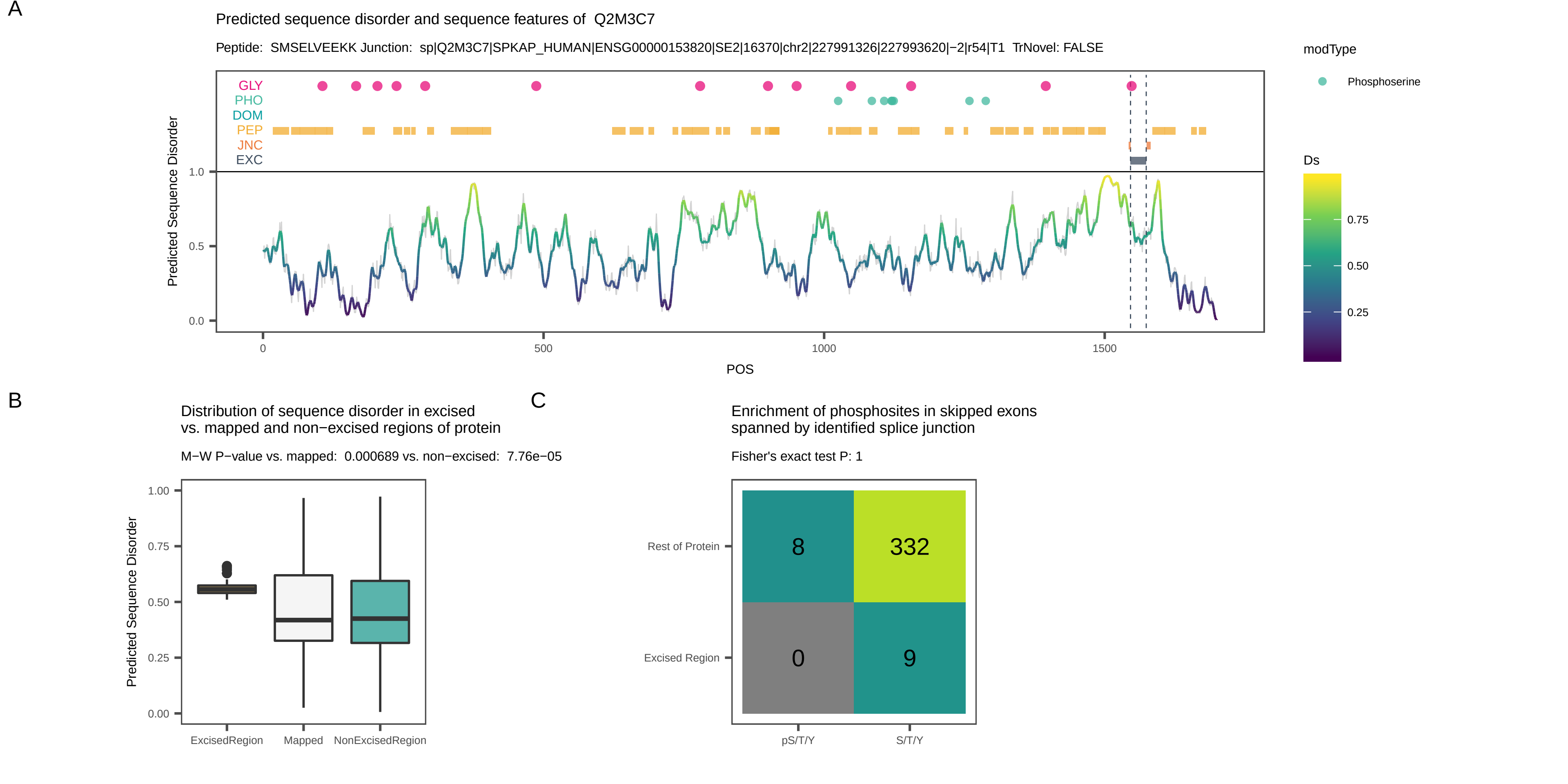
A
Predicted sequence disorder and sequence features of Q2M3C7
Peptide: SMSELVEEKK Junction: sp|Q2M3C7|SPKAP_HUMAN|ENSG00000153820|SE2|16370|chr2|227991326|227993620|−2|r54|T1 TrNovel: FALSE
GLY
PHO
DOM
PEP
JNC
EXC
Predicted Sequence Disorder
1.0
0.5
0.0
0
500
1000
1500
POS
modType
Phosphoserine
Ds
0.75
0.50
0.25
B
Distribution of sequence disorder in excised vs. mapped and non−excised regions of protein
M−W P−value vs. mapped: 0.000689 vs. non−excised: 7.76e−05
Predicted Sequence Disorder
1.00
0.75
0.50
0.25
0.00
ExcisedRegion
Mapped
NonExcisedRegion
C
Enrichment of phosphosites in skipped exons spanned by identified splice junction
Fisher's exact test P: 1
Rest of Protein
Excised Region
8
332
0
9
pS/T/Y
S/T/Y
